# A structural preview of aquaporin 8 via homology modeling of seven vertebrate isoforms

**DOI:** 10.1186/s12900-018-0081-8

**Published:** 2018-02-17

**Authors:** Andreas Kirscht, Yonathan Sonntag, Per Kjellbom, Urban Johanson

**Affiliations:** Division of Biochemistry and Structural Biology, Center for Molecular Protein Science, Department of Chemistry, Lund University, Box 124, SE-221 00 Lund, Sweden

**Keywords:** Homology modeling, Aquaporins, Selectivity, Ammonia, Molecular dynamics simulations

## Abstract

**Background:**

Aquaporins (AQPs) facilitate the passage of small neutral polar molecules across membranes of the cell. In animals there are four distinct AQP subfamilies, whereof AQP8 homologues constitute one of the smallest subfamilies with just one member in man. AQP8 conducts water, ammonia, urea, glycerol and H_2_O_2_ through various membranes of animal cells. This passive channel has been connected to a number of phenomena, such as volume change of mitochondria, ammonia neurotoxicity, and mitochondrial dysfunction related to oxidative stress. Currently, there is no experimentally determined structure of an AQP8, hence the structural understanding of this subfamily is limited. The recently solved structure of the plant AQP, *At*TIP2;1, which has structural and functional features in common with AQP8s, has opened up for construction of homology models that are likely to be more accurate than previous models.

**Results:**

Here we present homology models of seven vertebrate AQP8s. Modeling based on the *At*TIP2;1 structure alone resulted in reasonable models except for the pore being blocked by a phenylalanine that is not present in *At*TIP2;1. To achieve an open pore, these models were supplemented with models based on the bacterial water specific AQP, *Ec*AqpZ, creating a chimeric monomeric model for each AQP8 isoform. The selectivity filter (also named the aromatic/arginine region), which defines the permeant substrate profile, comprises five amino acid residues in *At*TIP2;1, including a histidine coming from loop C. Compared to *At*TIP2;1, the selectivity filters of modelled AQP8s only deviates in that they are slightly more narrow and more hydrophobic due to a phenylalanine replacing the histidine from loop C. Interestingly, the models do not exclude the existence of a side pore beneath loop C similar to that described in the structure of *At*TIP2;1.

**Conclusions:**

Our models concur that AQP8s are likely to have an *At*TIP2;1-like selectivity filter. The detailed description of the expected configuration of residues in the selectivity filters of AQP8s provides an excellent starting point for planning of as well as rationalizing the outcome of mutational studies. Our strategy to compile hybrid models based on several templates may prove useful also for other AQPs for which structural information is limited.

**Electronic supplementary material:**

The online version of this article (10.1186/s12900-018-0081-8) contains supplementary material, which is available to authorized users.

## Background

Fundamental intrinsic properties of a biological membrane are formed by its lipid composition, which can lead to unusual tight membranes, excluding certain solutes very efficiently [1]. Nonetheless, directed exchange of ions and uncharged molecules between compartments and dynamic permeant substrate selectivity of membranes require a repertoire of active transporters and passive channels, which can be readily regulated both in number and activity. In analogy with enzymes increasing the rate of a reaction by lowering the activation energy, channels facilitate diffusion across membranes by reducing an equivalent energy barrier for a permeating ion or uncharged molecule, from here on referred to as the substrate of the channel. In man, there are 13 channels belonging to the aquaporin (AQP) superfamily, which vary in their expression pattern and substrate selectivity [2]. Where some members of the superfamily specifically conduct water, others are also permeable to more bulky solutes. AQP8 is found in the inner mitochondrial membrane [3] and epithelial plasma membranes (reviewed in [4]). Well known to channel water and ammonia, more recent experiments in *Xenopus laevis* oocytes have added urea and glycerol to the substrate profile of some AQP8-paralogs in fish [5, 6]. Furthermore, heterologous expression of human AQP8 in yeast increased the sensitivity to H_2_O_2_-induced growth depression [7], and mitochondrial AQP8 knockdown in human cells showed reduced H_2_O_2_-release [8]. A similar substrate profile has been described for plant aquaporins belonging to the subfamily of tonoplast intrinsic proteins (TIPs), which are therefore understood as functionally related [7, 9, 10].

Despite localization studies and functional characterization, the biological function of AQP8s remains unclear. Situated in the inner mitochondrial membrane and being highly water permeable, AQP8 was suggested to facilitate rapid volume changes of mitochondria [11]. Accordingly, cholestatic liver disease was connected to reduced AQP8 expression resulting in decreased water permeability [12]. Additionally, AQP8 has been shown to conduct NH_3_ through planar bilayers [13]. While mammals have only one AQP8 isoform, some fishes are known to express up to three paralogs [14]. The fact that these gene duplications happened early in the evolution of fish AQP8s and that certain substitutions are conserved to allow formation of paralog groups, point towards a neo-functionalization for some of these proteins [14]. Differences in substrate profiles reported for different homologs of the AQP family can only be fully understood on the basis of the protein structures. Furthermore, the physiological role of AQP8 with its implications in diseases raises the demand for structural information of this protein family, potentially allowing knowledge-based drug design.

**Fig. 1:**
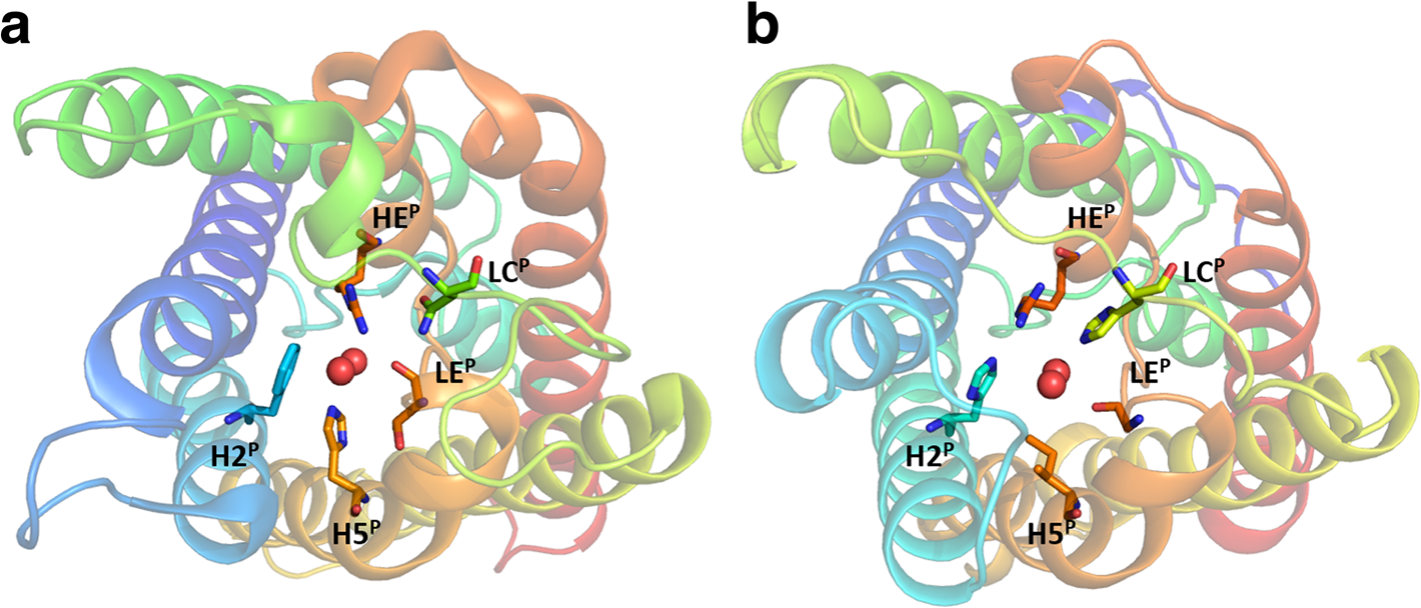
The five positions of the selectivity filter in *Ec*AqpZ and in *At*TIP2;1.The five amino acid residues shown in stick representation in *Ec*AqpZ (**a**) and *At*TIP2;1 (**b**) are used to describe the selectivity filter. The positions of these amino acids in the selectivity filter are named according to their localization in secondary structural elements: helix 2 (H2^P^), loop C (LC^P^), helix 5 (H5^P^), loop E (LE^P^), and helix E (HE^P^). Cartoon representation of the backbone with rainbow coloring, N- and C-terminal ends are blue and red, respectively. Oxygens of two water molecules in the selectivity filter are shown as red spheres

All aquaporin structures reported share a common fold and have been solved as homotetramers. Each monomer forms a functional pore by 6 transmembrane helices (helix 1–6) and two half-transmembrane helices (helix B and E). These two short helices point towards each other, forming a 7th pseudo transmembrane helix. The positive end of the macro-dipoles produced by these half helices are both facing towards the center of the membrane, each holding a conserved motif (Asn-Pro-Ala), the NPA box. This partial positive charge pose a significant energetic barrier for protons to overcome [15]. Most aquaporin isoforms additionally exclude protons by the positive charge of an arginine situated in a narrow part of the pore about 7 Å away from the membrane center towards the non-cytosolic side [16, 17]. As this region constitutes the highest energy barrier for various substrates, such as water, glycerol and ammonia [18], it is here referred to as the selectivity filter. The selectivity filter, also called the aromatic/arginine region, comprises four or five residues that interact directly with substrates in the pore [19–22]. The positions of these residues are termed according to the secondary structure they are embedded in (Fig. 1). The amino acid at the helix 2 position of the selectivity filter (H2^P^) typically has an aromatic sidechain that is oriented perpendicular to the radius of the pore. The helix 5 position (H5^P^) encompasses a histidine in water specific aquaporins but is replaced by a more hydrophobic residue in the TIPs. The amino acid residue in loop E (LE^P^) provides a carbonyl to the filter, which functions as hydrogen bond acceptor. This carbonyl is found in two distinct spatial orientations, and all available aquaporin structures can be grouped accordingly. In general, the carbonyl at LE^P^ of water specific aquaporins is hydrogen bonded to a residue from loop C (LC^P^), whereas it orients more towards H5^P^ in aquaporins of other subfamilies [22]. The non-hydrogen bonded carbonyl can interact more freely with pore substrates and may thereby compensate for a hydrophobic residue at H5^P^. Furthermore, in TIPs the residue at LC^P^, which is histidine in TIP2s, can also interact with pore substrates. From sequence alignments, it is clear, that for TIP3s a phenylalanine is located at the LC^P^ [22]. Beside the potential of hydrogen bonding to LE^P^ carbonyl and the interaction with a permeating molecule, the amino acid residue at LC^P^ can have an additional effect. Bulky sidechains at this position are able to shift the location of arginine in helix E (HE^P^). Depending on the residue at H2^P^ this shift is further stabilized by a hydrogen bond between the secondary nitrogen of arginine and the residue at H2^P^. By establishing the highest energy barrier for a permeating molecule, confining diameter and offering hydrogen bonding network, the selectivity filter is determining the substrate profile of the channel.

The distinctly different substrate profile of AQP8s compared to the well described water-specific aquaporins and glycerol facilitators enhances the interest in determining the molecular structure of an AQP8 and the determinants of its function. These structural determinants are difficult to derive based on known structures of these families in e.g. animals, because of sequence variation in the selectivity filter. Although human AQP8 analyzed with electron microscopy confirmed the formation of tetramers and 2D crystals [23], there is no solved structure of AQP8 available yet. So far, high resolution structures of mammalian AQP0, AQP1, AQP2, AQP4, and AQP5, which are classified as water specific aquaporins, have been solved [20, 24–27]. Additionally, a homology model of AQP9 was supported by a projection map at 7 Å [28]. Homology modeling approaches of a plant isoform functionally related to AQP8, the wheat (*Triticum aestivum*) *Ta*TIP2;1, have been applied using a water specific aquaporin (bovine AQP1) as a template [9]. Although the overall fold is conserved, the modelled selectivity filter did not comply with the TIP2 structure that was later solved (see below), demonstrating that comparative modeling of this region is strongly dependent on the used template. Sequence alignments of AQP8s combined with structural alignments of aquaporins of known structure has an isoleucine at H5^P^. Loop C can be aligned such that a phenylalanine is positioned at LC^P^, which would render the selectivity filter identical to TIP3s, aquaporins that are also expected to be permeable for ammonia and water [22, 23, 29]. Recently, our group solved the first structure of a functionally related protein from the TIP family (*At*TIP2;1 from *Arabidopsis thaliana*) and could thereby extend the view on the selectivity filter [22]. The new findings and revised alignments suggested that this structure should be a good template for homology modeling of AQP8.

Here, we explore this idea by modeling seven closely related AQP8 orthologs, for which common structural features are expected. The initial models are optimized using various techniques, and the final open monomeric models are analyzed structurally. For one of the seven monomeric models, a tetrameric model is generated, and the stability of the *At*TIP2;1-like selectivity filter is validated by molecular dynamics (MD) simulations.

## Results

### Modeling and stereochemical evaluation

**Fig. 2:**
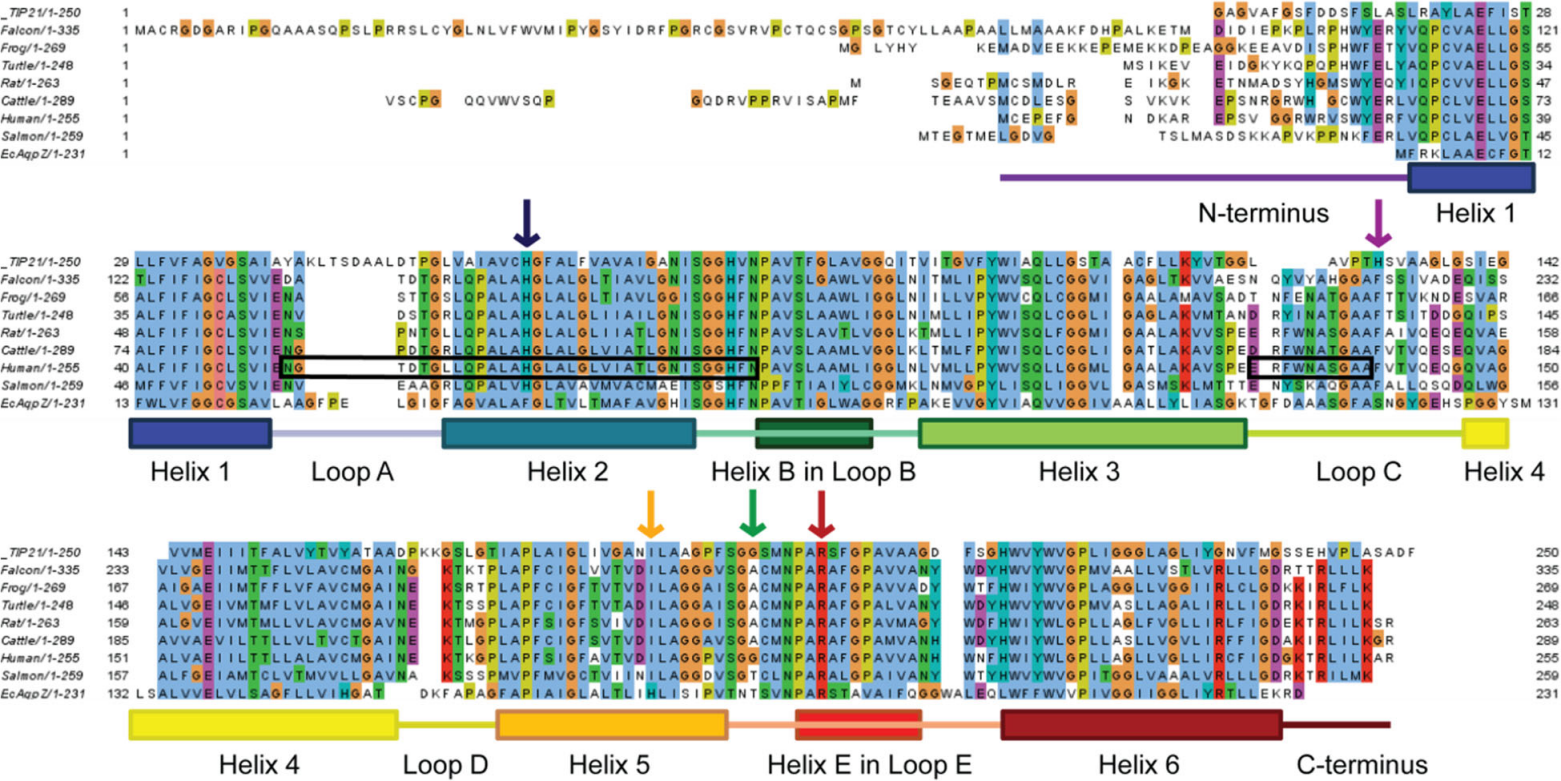
Alignment of seven vertebrate AQP8s together with the modeling templates *At*TIP2;1 and *Ec*AqpZ. Secondary structures as modelled in *Hs*AQP8 are depicted beneath the multiple sequence alignment. The amino acids are colored according to their chemical properties. Residues that are part of the selectivity filter are depicted by vertical arrows: H2^P^ – black, LC^P^ – purple, H5^P^ – yellow, LE^P^ – green, HE^P^ – red. Black boxes mark regions in *Hs*AQP8 that are modeled based on *Ec*AqpZ. See also Table 1 and Additional file 2: Figure S2

**Fig. 3:**
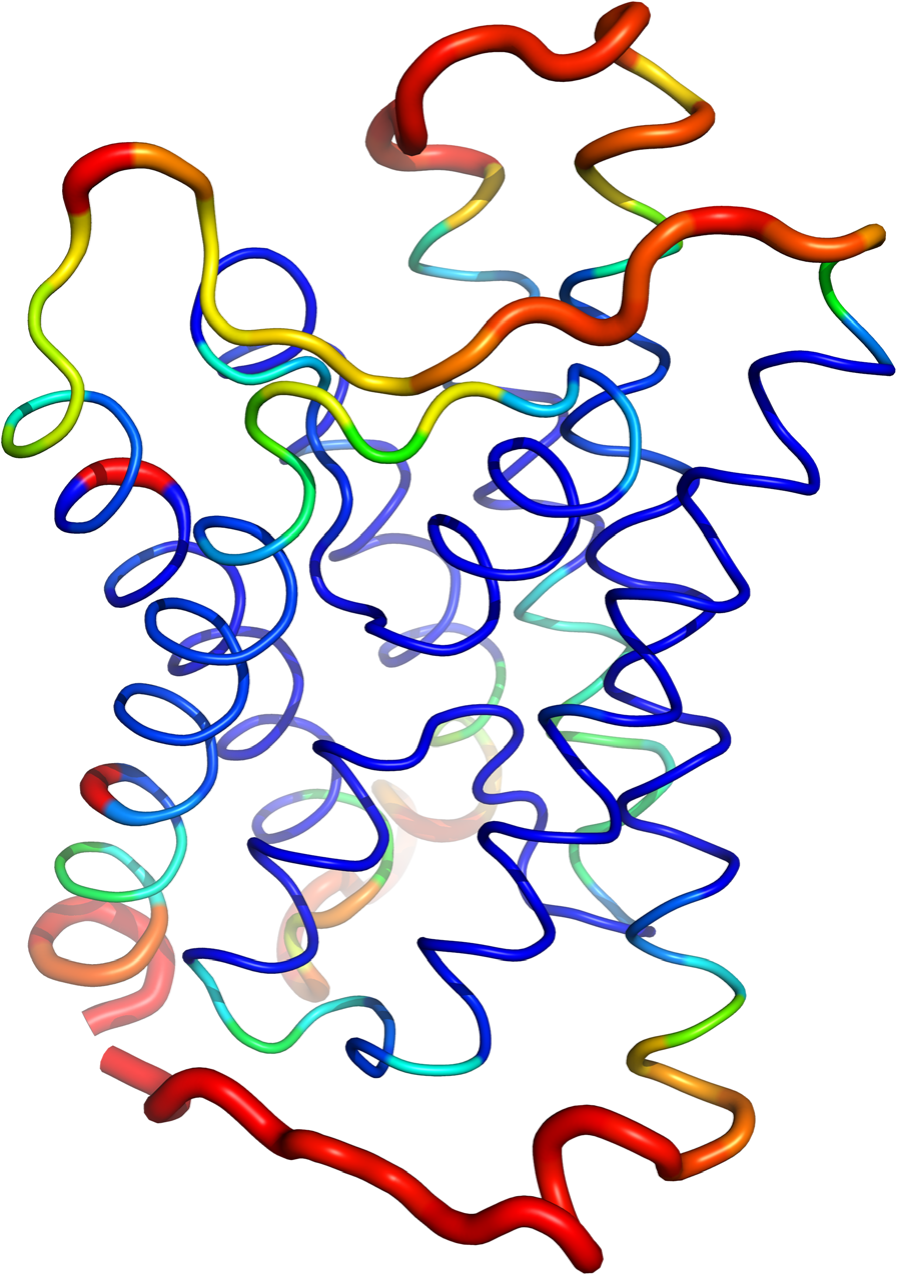
Structural conservation of C_α_ positions in AQPs mapped onto the structure of *At*TIP2;1. The result of structural alignment of 49 deposited AQP structures from the PDB is indicated on the *At*TIP2;1 structure. Thin (blue) putty indicates high degree of structural conservation, while thick (red) putty indicates low degree of structural conservation. In general, transmembrane helices are highly conserved, while loops are more variable. The longest loop in the upper part of the picture, loop C, has a high degree of variance at the N-terminal end (right), while the LC^P^ (middle) and the fold at the C-terminal region (left) before helix 4 is more conserved. PDB IDs are provided in Additional file 6

In this study AQP8s from seven vertebrates, namely from man (*Homo sapiens, Hs*AQP8), cattle (*Bos taurus, Bt*AQP8), rat (*Rattus norvegicus, Rn*AQP8), falcon (*Falco peregrinus, Fp*AQP8), turtle (*Chrysemys picta bellii, Cp*AQP8), frog (*Xenopus tropicalis, Xt*AQP8), and salmon (*Salmo salar, Ss*AQP8b), were independently modelled.The initial homology modeling was based on *At*TIP2;1, for which a multiple sequence alignment with the modelled AQP8 isoforms is presented in Fig. 2. The selected AQP8s are relatively closely related and present a pairwise amino acid sequence identity to *Hs*AQP8 ranging from 47.8% for *Ss*AQP8b to 81.6% for *Bt*AQP8 (Additional file 1: Figure S1). Their sequence identity with *At*TIP2;1 is at least 30% (*Ss*AQP8b). A structural alignment of representative solved aquaporin structures indicates parts that are more likely to be modelled correctly due to conservation (Fig. 3). The longest loop, loop C, contains two sections that are structurally more conserved than the rest of this loop: the middle region at LC^P^ and the C-terminal region before helix 4. Using this as a guide, alignment of the LC^P^ residue can be done with higher confidence. With prior structural knowledge revealing the possibility of an aromatic residue at this position in aquaporins [22], the choice of phenylalanine at LC^P^ of AQP8s seems very reasonable. Initially, all the AQP8 models solely based on *At*TIP2;1 were unexpectedly blocked by another phenylalanine that is conserved among AQP8s (F85 in *Hs*AQP8) but corresponding to a valine (V82) in *At*TIP2;1. As it has been shown for several of these channels that they are highly permeable to water and ammonia, it is reasonable to expect that the pore of the model should be wide enough to allow for passage of these substrates [13, 23]. By sequence alignment and comparison of structures a corresponding phenylalanine in a spatial orientation that supports an open pore, was identified in AqpZ from *Escherichia coli*. To realize a functional pore, additional AQP8 models were therefore created using *Ec*AqpZ as template. The resulting models showed a slight shift of helix 1 and 2 that leave enough space to accommodate the phenylalanine between them, leading to an unblocked pore. A monomeric hybrid model of each AQP8 isoform was then created by combining different of parts of the two models generated from the different templates (Table 1, and Additional file 2: Figure S2). Additionally, for one of the models (*Ss*AQP8b) another phenylalanine specific for this paralog (F95, following the rare NPP motif analyzed below) had to be manually rotated to clear the pore.

**Table 1:** Composition of hybrid models compiled from homology models based on *At*TIP2;1 and *Ec*AqpZ

AQP8	Accession	*At*TIP2;1 modelled	*Ec*AqpZ modelled
Human 1–255	AAH40630.1	12–52; 87–124; 139–245	53–86; 129–138
Cattle 1–289	AAI16017.1	46–86; 120–158; 172–276	87–119; 159–171; 272–281
Rat 1–263	NP_062031.1	20–60; 94–132; 146–253	61–93; 133–145; 254–255
Turtle 1–248	XP_005289228.1	7–31; 81–240	32–80; 241–242
Frog 1–269	NP_001107728.1	28–74; 102–261	75–101; 262–263
Falcon 1–335	XP_013151085.1 (=79–335)	94–125; 168–327	126–167; 328–329
Salmon (AQP8b) 1–259	NP_001167386.1	18–57; 96–152; 160–251	58–95; 153–159

**Fig. 4:**
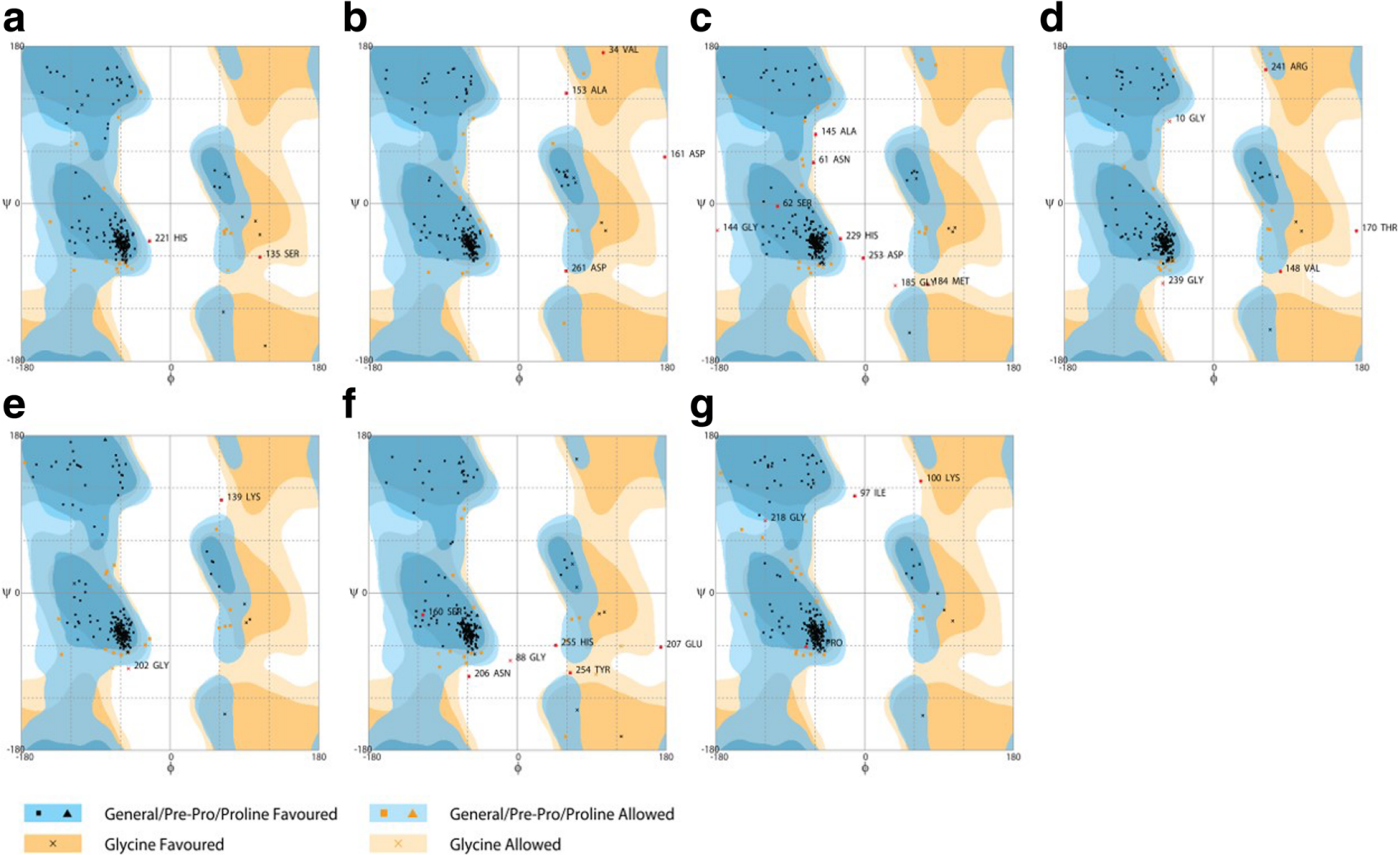
Ramachandran plots of AQP8 models. All outliers are identified by position and three letter code. For a summary see Table 2. **a** *Hs*AQP8. **b** *Xt*AQP8. **c** *Rn*AQP8. **d** *Cp*AQP8. **e** *Ss*AQP8b. **f** *Bt*AQP8. **g** *Fp*AQP8. The plots were generated with RAMPAGE [31]

The final monomeric AQP8 homology models were evaluated for stereochemical quality with the QMEAN-server [30], and Ramachandran plots were generated using RAMPAGE [31]. The Ramachandran plots are shown in Fig. 4, and further details are given in Table 2. The Ramachandran outlier residues all lay within loop sections, which are the structurally most variant part of the aquaporins. However, the high sequence similarity at important positions between the modelled isoforms and *At*TIP2;1 and the consistency, as well as the reasonable stereochemical quality of the final models, justify drawing some conclusions.

**Table 2:** Summary of Ramachandran plots for AQP8 models

Number of residues in:	Cattle	Falcon	Frog	Rat	Turtle	Human	Salmon
Favored region	211 (90.2%)	208 (89.7%)	212 (90.6%)	204 (87.2%)	210 (89.7%)	212 (91.4%)	210 (90.5%)
Allowed region	17 (7.3%)	20 (8.6%)	18 (7.7%)	22 (9.4%)	19 (8.1%)	18 (7.8%)	20 (8.6%)
Outlier region	6 (2.6%)	4 (1.7%)	4 (1.7%)	8 (3.4%)	5 (2.1%)	2 (0.9%)	2 (0.9%)

### Pore and selectivity filter

**Fig. 5:**
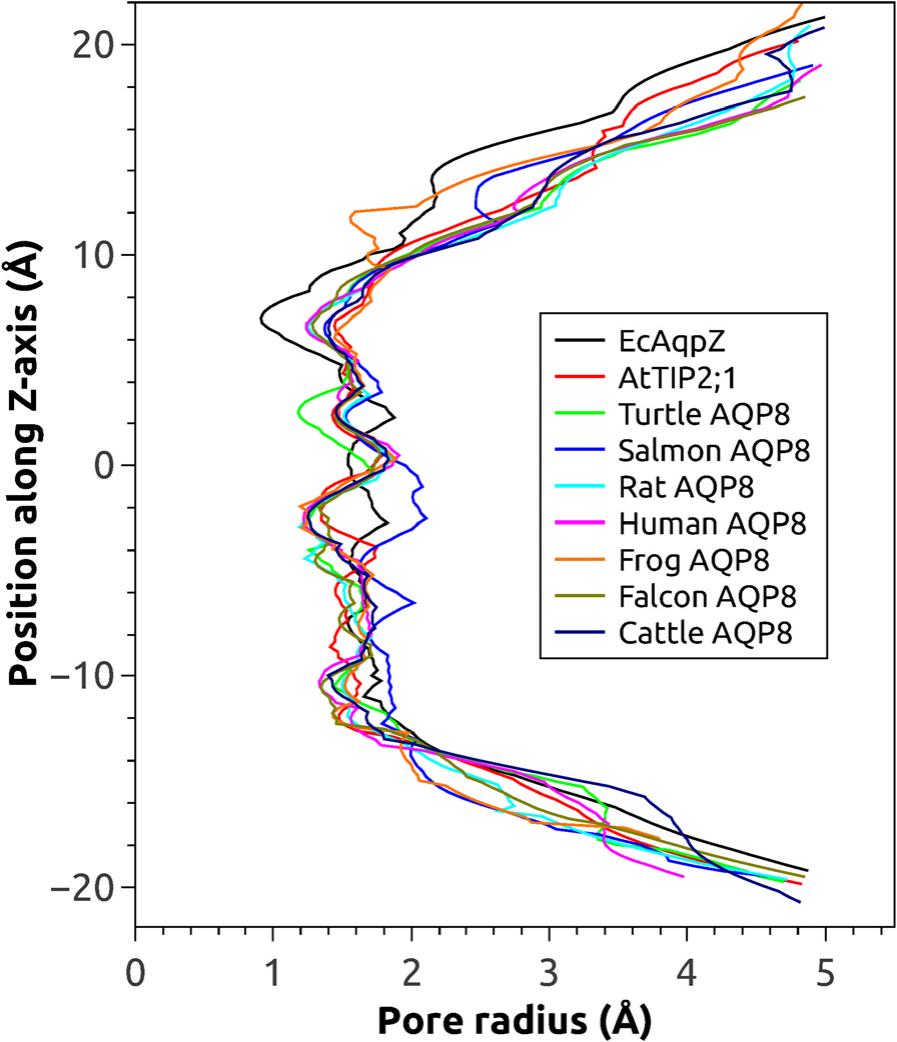
Comparison of pore radii along the axis of the pore. Similar to *At*TIP2;1 (red), most of the models have a rather uniform radius throughout the pore. The radii of the AQP8 models are particularly homogeneous at the selectivity filter (around 7 Å on the z axis) where they deviate from water specific AQPs like *Ec*AqpZ (black) which have their most narrow region here. Interestingly the salmon AQP8 (*Ss*AQP8b; blue) is wider at the lower NPA region (around − 2 Å on the z axis)

In the final models, the diameter of the pore is relatively wide and fairly uniform along the axis of the pore as compared to water specific AQPs where the selectivity filter clearly constitutes the most narrow region (Fig. 5). Substrate profiles of aquaporins are believed to be defined by their selectivity filter, and residues in this region are generally well conserved within each subgroup or even within a whole subfamily. The dimensions of the pore in the AQP8 models are very similar to *At*TIP2;1 [22], and although there is some variation between the models as discussed later, the selectivity filter region is the least variable part in the modelled pores.

**Fig. 6:**
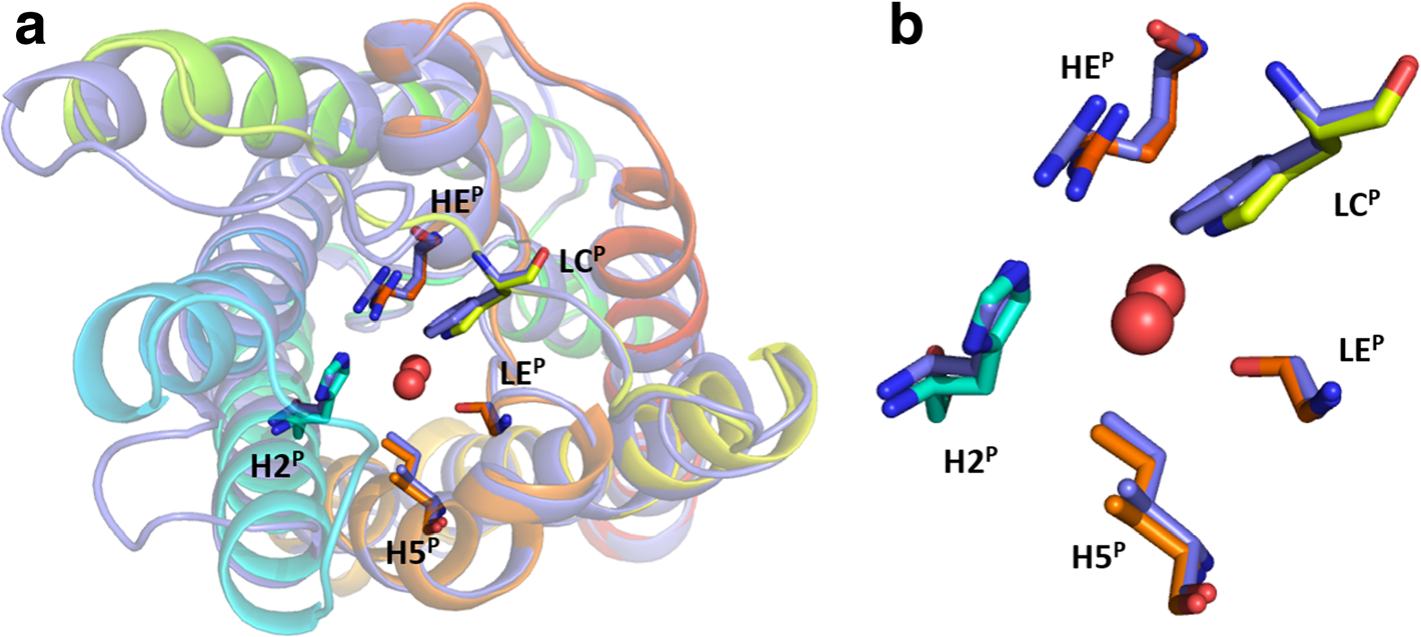
Selectivity filter of the modelled human AQP8. **a** The monomeric model of *Hs*AQP8 (slate blue) is aligned to the template *At*TIP2;1 (rainbow). The residues in the selectivity filters are shown in stick representation. **b** Close up of the residues in the selectivity filters. Selectivity filter of *Hs*AQP8 and *At*TIP2;1 consists of H66 (H2^P^), F139 (LC^P^), I192 (H5^P^), G201 (LE^P^), R207 (HE^P^) and H63 (H2^P^), H131 (LC^P^), I185 (H5^P^), G194 (LE^P^), R200 (HE^P^), respectively. Oxygens of two water molecules in the selectivity filter are shown as red spheres

The models confirm that the histidine in H2^P^, although it is located in a region of the hybrid model that was initially modelled on *Ec*AqpZ, can be fitted in a similar position as in *At*TIP2;1 where it stabilizes the arginine in HE^P^ in a *At*TIP2;1-like orientation (Fig. 6, Additional file 3: Figure S3). Furthermore, the isoleucine at H5^P^ is situated in an identical spatial location in the AQP8 models and in *At*TIP2;1. The LE^P^ residues, contributing their carbonyls to the selectivity filter, are consistently small in both AQP8s and *At*TIP2;1. While LE^P^ in *Hs*AQP8 is glycine as in TIP2s, this residue is replaced by alanine in all but salmon AQP8, occupying essentially the same space. In *Ss*AQP8b, LE^P^ is occupied by the slightly larger threonine residue, as in *Ec*AqpZ, but the orientation of its carbonyl is not deviating from those in other AQP8 models. This may suggest that it is sufficient with any small residue at this position to achieve the same substrate profile. None of the modelled LE^P^ carbonyls can form a hydrogen bond to the side chain of the residue at LC^P^ since this is a phenylalanine in all the models. As a result, all LE^P^ carbonyls reside in a relaxed position, like in the structures of all other AQPs that are not limited to water as a substrate [22]. A further effect of the phenylalanine at LC^P^ is the stabilization of the arginine at HE^P^ in an *At*TIP2;1-like position. Where arginines at HE^P^ in water specific aquaporins and aquaglyceroporins are oriented towards the pore, phenylalanine in AQP8s shifts the arginine towards the H2^P^ histidine. There it probably hydrogen bonds to the imidazole group of the histidine, lowering the pK_*a*_ of the latter, as has been suggested for *At*TIP2;1 [22]. To assure that this hydrogen bond can be formed, the protonation state and the rotamer of H2^P^ histidine were modelled accordingly. In return, the pK_*a*_ of this histidine is predicted by PROPKA3 [32] to be significantly lowered (e.g. 4.92 in *Bt*AQP8). The potential of arginine to get so close to the histidine to modify its acidity is depending on its spatial constrains and other stabilizing interactions (see Discussion).

### Structural analysis of the NPP motif

**Fig. 7:**
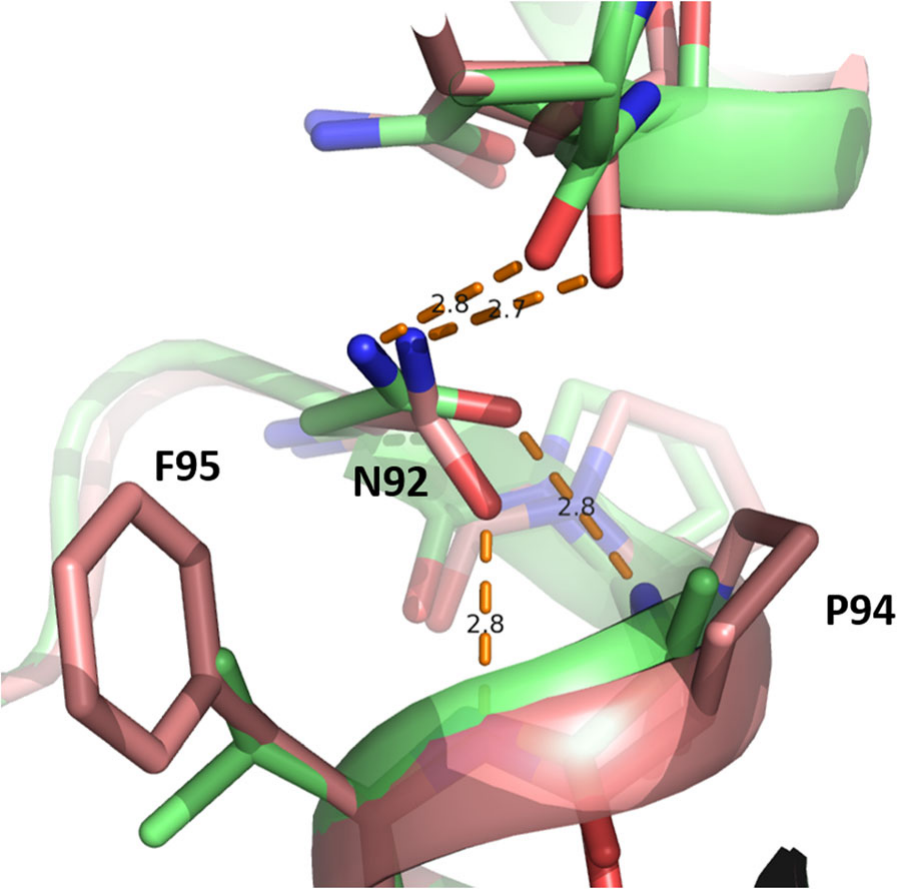
Rare NPP motif in salmon AQP8 changes the hydrogen bonding and widens the pore. In spite of the unusual NPP motif, the overall fold of the *Ss*AQP8b model (salmon) is similar to the template *At*TIP2;1 (green) harboring the canonical NPA motif. Nevertheless, the second proline in the NPP motif of *Ss*AQP8b (P94) eliminates a hydrogen bond partner of the asparagine (N92), which instead turns to the next amide of the backbone (F95). The altered orientation of the asparagine widens the pore and potentially optimizes the polar interactions with the permeant substrate

The NPA boxes cap the half-helices, while the positive charge of the macro dipoles is focused at the amide of the asparagines in the NPA motifs. Their multiple functions including proton exclusion, connection of the two half helices via hydrogen bonding, and lining of the pore with hydrogen bond donors are likely reasons for the high degree of conservation of this motif. Yet, there is a huge variety of deviating sequences known at this region. The MIP modeling database MIPModDB [33] currently lists 1507 AQPs, presenting NPA as motif 2502 times, which is much less than expected from the conserved dual symmetry of AQPs. Besides NPA, NPS and NPP are found in fish AQP8s, the latter being most unusual among aquaporins. Interestingly, two salmon AQP8 paralogs have NPP in the first box, while *Ss*AQP8aa1 has only canonical NPA motifs [6]. Do these variations in sequence lead to functional differences between the isoforms? In oocyte experiments, all salmon paralogs showed water and urea permeability, but the NPP containing proteins were in addition permeable to glycerol [6]. From our models, we can conclude that the second proline of NPP (P94 in *Ss*AQP8b) render the secondary structures unchanged, although the backbone may be more constrained compared to AQPs with an alanine at this position (Fig. 7). Notably, all NPP motifs in the non-redundant NCBI database (nr) that were found by a BLAST search with *Ss*AQP8b are followed by phenylalanine replacing the conserved smaller hydrophobic residue (V89 in *Hs*AQP8) immediately after the first NPA motif (data not shown). The backbone constraints could evoke that this phenylalanine (F95 in *Ss*AQP8b) is kept out of the pore. The modelled conformation is further substantiated by a smaller hydrophobic residue lining the pore (V168 in *Ss*AQP8b instead of isoleucine in the other models), leaving a pocket for the more bulky aromatic sidechain. Furthermore, the second proline of the NPP motif (P94 in *Ss*AQP8b) appears to change the hydrogen bonding of the asparagine in the motif (N92 in *Ss*AQP8b) towards the amide of F95. The resulting rotation of the asparagine widens the pore in the NPA region, as evident in the pore profile (Fig. 5).

### Surface electrostatics

**Fig. 8:**
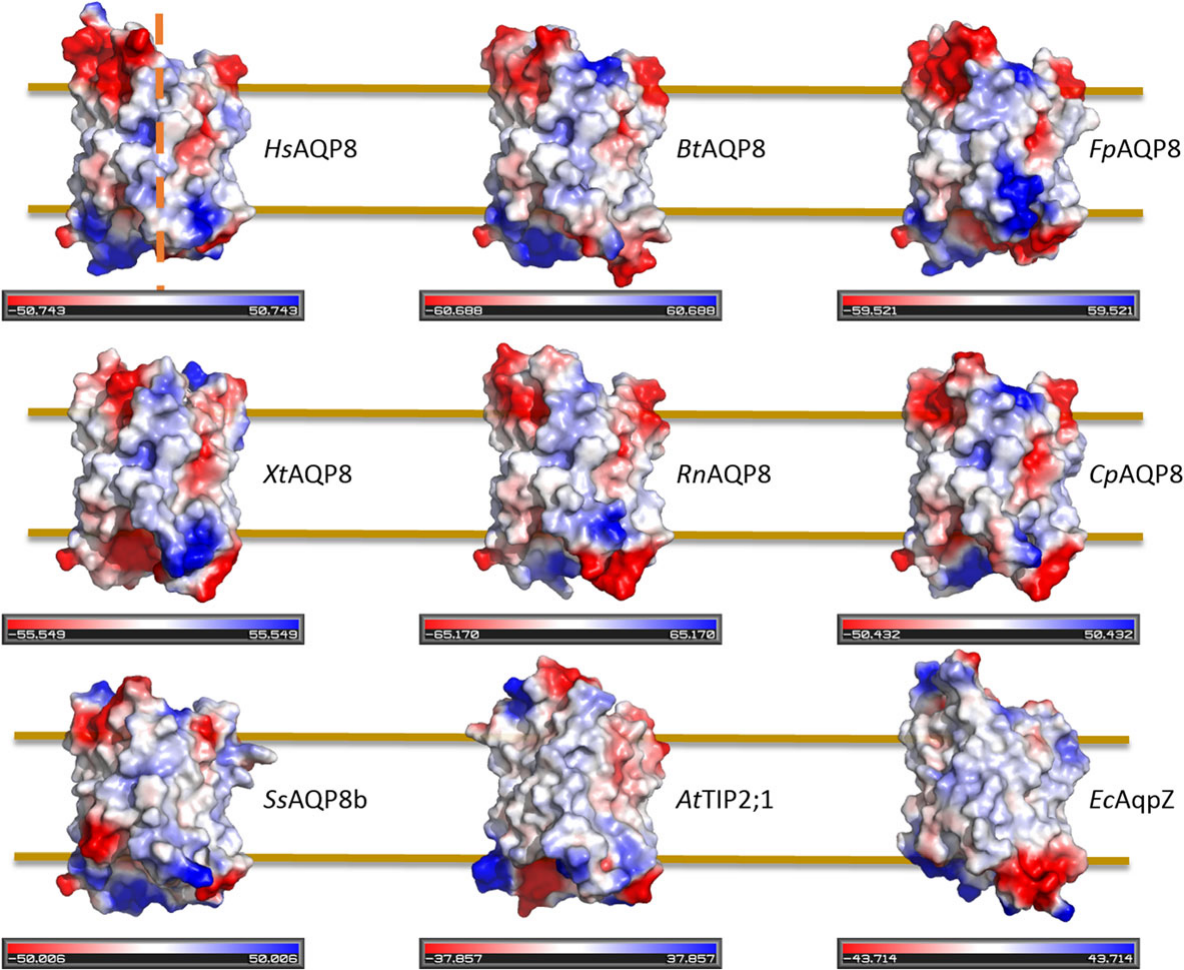
Surface electrostatics of AQP8 models. The slightly tilted side views show clear differences in potential between the C loop side (top) and the opposing side. Red areas represent relatively negative surfaces, while blue are positive. The approximate position of the membrane is shown by vertical lines and the central axis of the tetramer (dashed line), is indicated on *Hs*AQP8. Note the negative surface deep in the membrane on the right of all models except the salmon AQP8b. This charge is due to an aspartate which corresponds to an asparagine in *At*TIP2;1 and *Ss*AQP8b, hence the aspartate is more likely to be in a protonated state in this hydrophobic environment. The vacuum electrostatics for individual monomers were generated in PyMOL [40] and are only for relative comparisons within the individual monomer as the scale varies between monomers

When comparing *At*TIP2;1, which resides in the vacuolar membrane, i.e. the tonoplast, with an aquaporin located in the plasma membrane (*So*PIP2;1), a difference of the non-cytosolic surface was pointed out [22]. The more acidic surface on the luminal side of *At*TIP2;1 was connected to the lower pH in the vaccuole. Although the mitochondrial intermembrane space may only have a slightly lower pH than the cytosol depending on the metabolic state, its pH of 6.8 is significantly more acidic (around 0.8 pH units) than the mitochondrial matrix [34, 35]. We hypothesized that this pH difference is reflected in the electrostatic potentials of the opposing surfaces of AQP8s. The overall picture is that the surface close to the selectivity filter is more negative than the side harboring the N and C termini (Fig. 8).

In Fig. 8 all modelled isoforms except *Ss*AQP8b display a negative surface in the middle of the membrane-spanning region due to an aspartate (D191 in *Hs*AQP8) preceding the residue in H5^P^. However, the hydrophobic milieu and the observation that the corresponding residue in *Ss*AQP8b and in *At*TIP2;1 is a polar but uncharged asparagine (N184 in *At*TIP2;1) suggest that this aspartate is most likely protonated, hence uncharged in this setting.

### Validation by MD simulation

Apart from examining the stereochemical qualities of the models, they may be evaluated by studying their stability in MD simulations. The simulation allows the model to equilibrate as a tetramer in an adequate milieu and is expected to resolve local unfavorable states, resulting in a more relevant and stable conformation. Similar to the majority of the solved AQP structures, the homotetrameric *At*TIP2;1 structure was solved using a 4-fold non-crystallographic symmetry, thus treating all monomers as identical. We used the symmetry operator from the *At*TIP2;1 structure to form a homotetramer of the monomeric *Hs*AQP8 model. This operation restricted the model to the same tetrameric packing, including the distance of the monomers to the symmetry axis and thereby to each other. Considering that interface residues of the monomer were energy minimized in vacuum, they were not expected to be in the right orientation for a tight interaction between monomers. To avoid clashes and to optimize the packing, energy minimizations of the tetramer were done in a stepwise fashion with a gradual release of constraints.

**Fig. 9:**
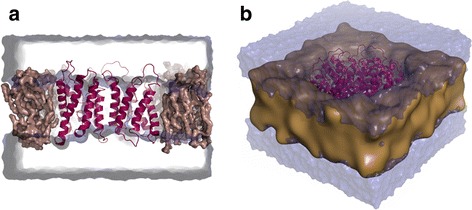
MD simulation setup of tetrameric *Hs*AQP8 in membrane. Simulation setup after equilibration of waters (blue), ions and lipids (POPC lipids colored in sand) to the protein *Hs*AQP8 model (red). **a** Side view showing two monomers of the tetramer inserted in the POPC membrane, and the water boundary (blue/grey). The slightly protruding loop C is visible at the top. **b** Unit cell displayed from above

**Fig. 10:**
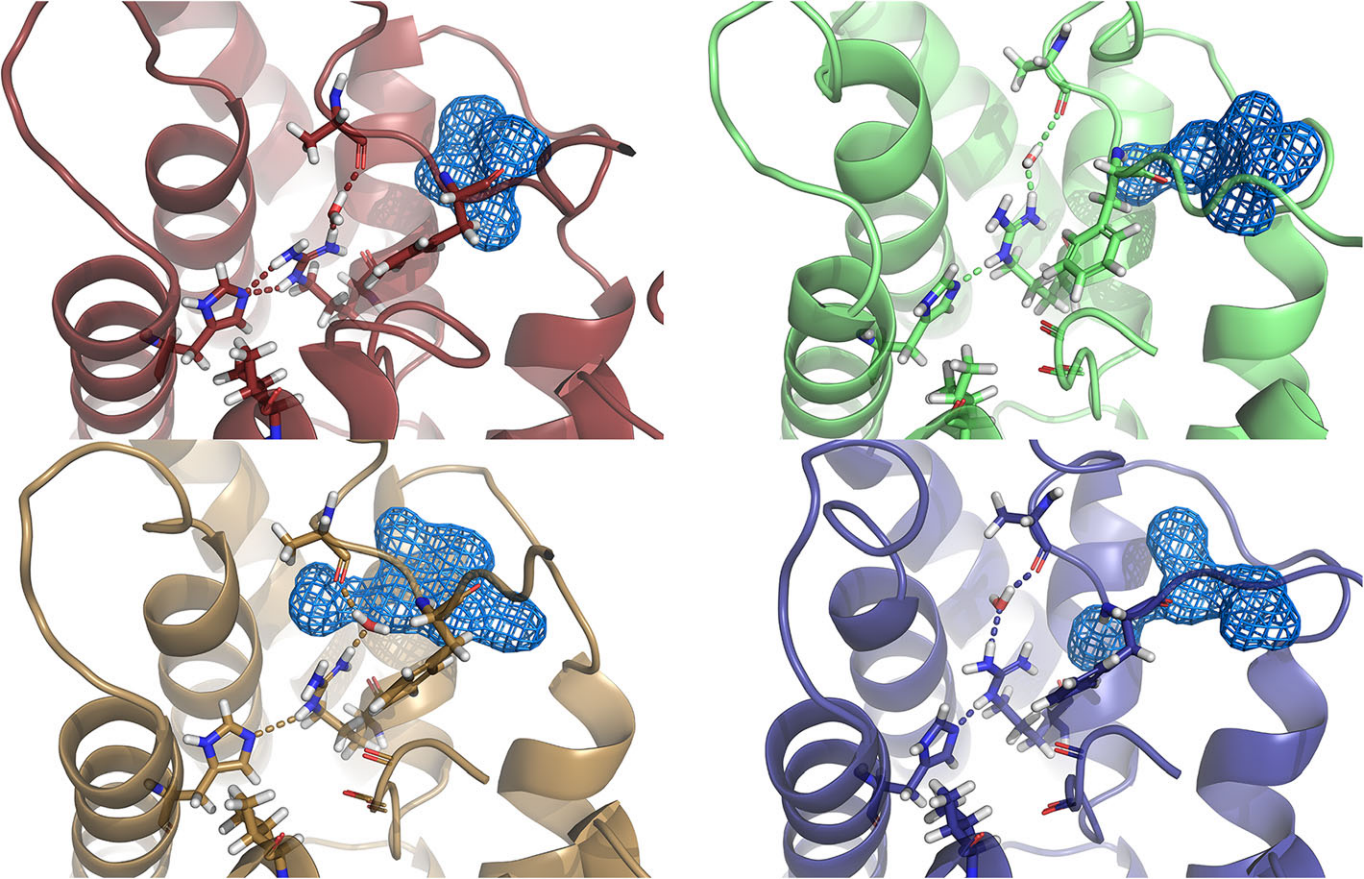
The selectivity filter of *Hs*AQP8 remains *At*TIP2;1-like during the MD simulation. The selectivity filter of each of the four monomers of the tetramer in the last frame of the 3 ns simulation, the monomers are aligned to facilitate comparisons. The residues of the selectivity filter, H66 (H2^P^), F139 (LC^P^), I192 (H5^P^), G201 (LE^P^), R207 (HE^P^) as well as A137 in loop C are shown in stick representation together with the carbonyl oxygens of G200 and C202 lining the pore. R207 is stabilized in an *At*TIP2;1-like position via hydrogen bonds (dashed lines) to H66 and to A137 of loop C via a water molecule, which has a variable position and is exchanged occasionally (Additional file 5: Movie S1). The orientation of the carbonyl of G201 (LE^P^) mainly points to the centre of the pore. The side pore remains more or less water filled during the simulation; the blue mesh marks the surface of water molecules in the last frame


A pre-simulation setup of the *Hs*AQP8 tetramer equilibrated in a lipid membrane can be seen in Fig. 9. Interestingly, it was required that an aspartate (D191 in *Hs*AQP8, corresponding to N197 in *Ss*AQP8b), was protonated and uncharged to achieve a stable setup. This is consistent with its local environment being hydrophobic, and with our evaluation of the surface electrostatics of the monomer as well as the conservation pattern among AQP8s and *At*TIP2;1 as mentioned above. The C_α_ RMSD relative to the starting tetrameric model did stabilize during the 3 ns MD simulation, indicating that a conformational equilibrium had been reached during the run (Additional file 4: Figure S4). Visual inspection of the *Hs*AQP8 tetramer during the 3 ns MD simulation supports that the pores of the four monomers stay open during the simulation and that the selectivity filter remains in an *At*TIP2;1-like configuration (Fig. 10). Importantly, the interaction between the histidine (H66) at H2^P^ and the arginine (R207) at HE^P^ is stable over time, and a *At*TIP2;1-like hydrogen bond network that connect the arginine to a carbonyl of loop C is formed and persists, although the mediating water molecules are moving and exchanged in the course of simulation (Additional file 5: Movie S1). Similarly, the putative side pore remains water-filled although the positions of water molecules are not static. Thus, MD simulations of the *Hs*AQP8 tetramer support that the model is stable and that the selectivity filter after the simulation conforms to that of monomeric model of the other AQP8s. Hence, it is likely that these AQP8 models provide a valid structural hypothesis that can be exploited in future work.

Additional file 5:**Movie S1.** The *At*TIP2;1-like selectivity filter in *Hs*AQP8 and stable water molecules. The movie is showing the 30% least mobile water molecules at one of the monomeric pores in the tetrameric model of *Hs*AQP8 during the 3 ns MD simulation. Four of the residues of the selectivity filter, H66 (H2^P^), F139 (LC^P^), I192 (H5^P^), and R207 (HE^P^) are shown in stick representation, together with A137 in loop C. The selectivity filter maintains an open *At*TIP2;1-like conformation, where R207 is stabilized in an *At*TIP2;1-like position via hydrogen bonds to H66 and to the carbonyl A137 of loop C via two water molecules consecutively (shown as Van der Waals spheres). The movements of the protein and water molecules are averaged over several ps to smoothen the movie. The color of water molecules is proportional to their momentary speed, from red for the fastest to deep blue for the slowest. (MP4 1124 kb)


## Discussion

A relevant model is a prerequisite for structural based discussions on channel properties. However, achieving a high resolution structure of a eukaryotic membrane protein may be both laborious and time demanding, if at all possible. In the absence of an experimentally determined structure, homology models can provide a conjecture of the most reasonable three dimensional structure of the protein of interest. Based on sequence alignments we have hypothesized that *At*TIP2;1 should be a good template for homology modeling of selected AQP8s, since they appear to share identical residues in at least three of the five positions of the extended selectivity filter, have a small residue in LE^P^, and can be aligned with an aromatic residue at the LC^P^ position of the filter [22]. It should be noted that employing template based methods does not automatically yield reasonable structures. For example, Swiss Modeller did not return a model for AQP8 from tardigrade (*Milnesium tardigradum*), which has a sequence identity of 21% to *At*TIP2;1, as a loop could not be aligned well enough (data not shown). Furthermore, the model of AQP8 from parakeet (*Melopsittacus undulates*) was discarded, since not all helices could be modelled, resulting in an insufficiently stable structure.

To achieve an open pore, structural information from *Ec*AqpZ was incorporated in the presented models of AQP8. This strategy of creating a composite model by combining elements from different structures is a general approach that might be useful also for other AQPs lacking a 3D structure. Our models confirm that the studied AQP8s are likely to have an *At*TIP2;1-like extended selectivity filter. A prominent feature of this filter is the specific orientation of the arginine at HE^P^, engaged in an interaction with the histidine at H2^P^ and additionally constrained by the phenylalanine at LC^P^ fitted next to the arginine. Notably, the automatically built *Hs*AQP8 model currently provided in the MIP modeling database MIPModDB [33] is not taking the recent *At*TIP2;1 structure into account and is fundamentally different from the models presented here, as it is not placing the arginine at HE^P^ in a *At*TIP2;1-like orientation. Arginine and phenylalanine are able to stabilize each other by cation-π interaction. The strength of this interaction is depending on the distance between the guanidinium group of arginine and the aromatic ring and their relative orientation. Interestingly, the proximity of phenylalanine to arginine could cause a deformation of the π-electron clouds, which in turn could have an effect on interactions between phenylalanine and pore solutes.

**Fig. 11:**
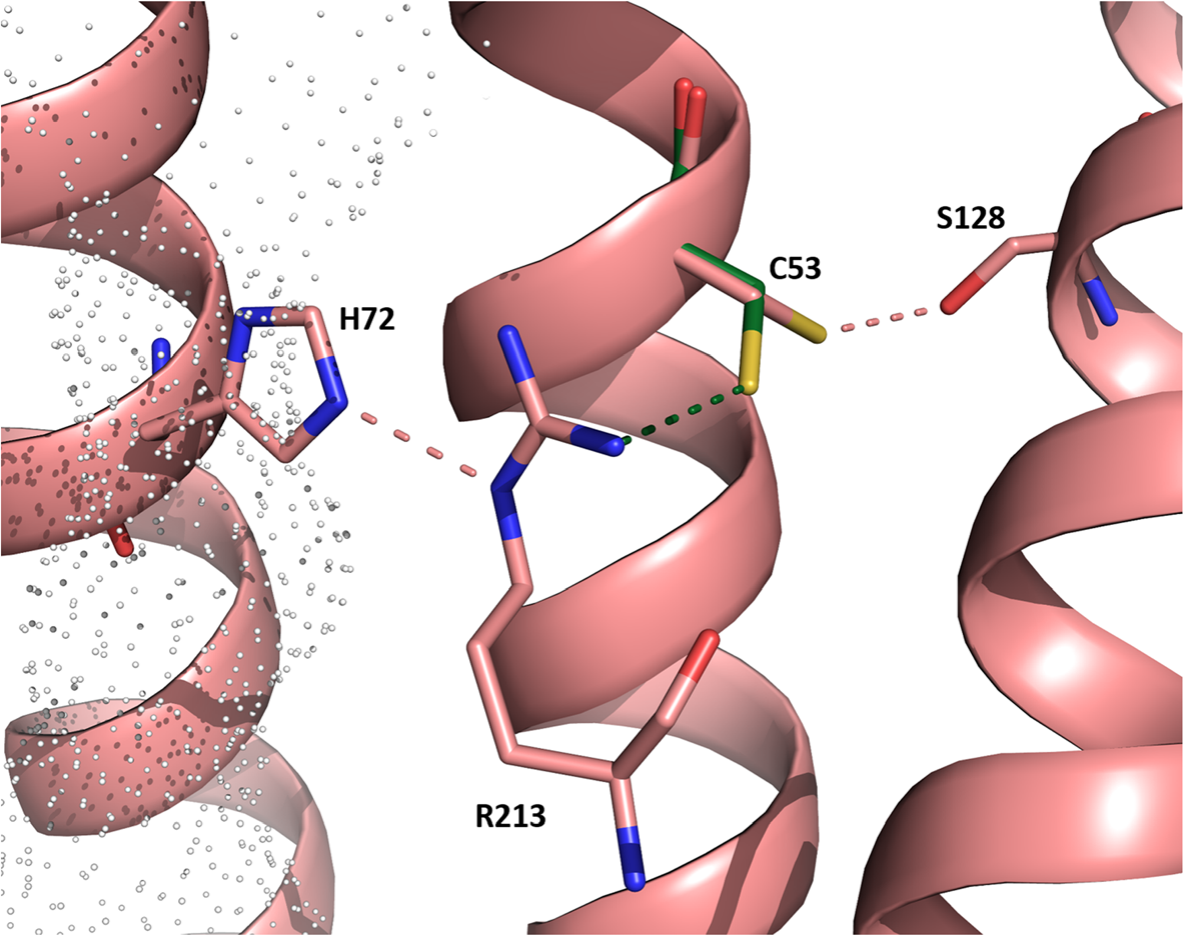
Alternative cysteine rotamers at the selectivity filter of salmon AQP8. Cysteine 53 is modelled in two alternative conformations. The rotamer colored in green is turned towards arginine 213 at HE^P^ with a distance of 3.3 Å. If not oriented towards arginine 213, cysteine 53 can hydrogen bond to a serine (S128) in helix 3. Hydrogen bonds are depicted as dashes, and the pore is contoured by a dotted surface. The histidine at H2^P^ (H72) stabilizes the position of arginine 213 by hydrogen bonding to the secondary ketimine

AQP8s may utilize an additional way to stabilize arginine in a *At*TIP2;1-like position. On the side of the arginine not facing the pore, some AQP8s harbor a cysteine in helix 1. In our automatically optimized models, this potential hydrogen bond partner is oriented away from the arginine at HE^P^. This orientation and an alternative rotamer are exemplified for *Ss*AQP8b cysteine 53 (C53) in Fig. 11. All three salmon AQP8 paralogs (aa1, ab, and b) are, as typical for aquaporins, susceptible to blocking by mercury, although *Ss*AQP8aa1 exhibits a much lower sensitivity [6]. As C53 (*Ss*AQP8b) is close to the HE^P^ arginine, binding of mercury could potentially block the pore indirectly via displacement of the arginine. Interestingly, a difference between *Ss*AQP8aa1 and the other two paralogs is an alanine that replaces serine 128 (S128 *Ss*AQP8b), which hydrogen bonds to C53 in our model.

It is known that arginines can change the pK_*a*_ of close cysteines [36]. This may influence the susceptibility of C53 to oxidation. An oxidized cysteine might, in turn, change the location of the arginine at HE^P^ and thereby modify the permeability of the selectivity filter. If an oxidizing agent, e.g. H_2_O_2_, can access this cysteine (C53 in *Ss*AQP8b), it may, therefore, regulate the aquaporin function. Interestingly, there are recent results supporting that stress can regulate the permeability of AQP8 via the oxidation state of this cysteine [37].

**Fig. 12:**
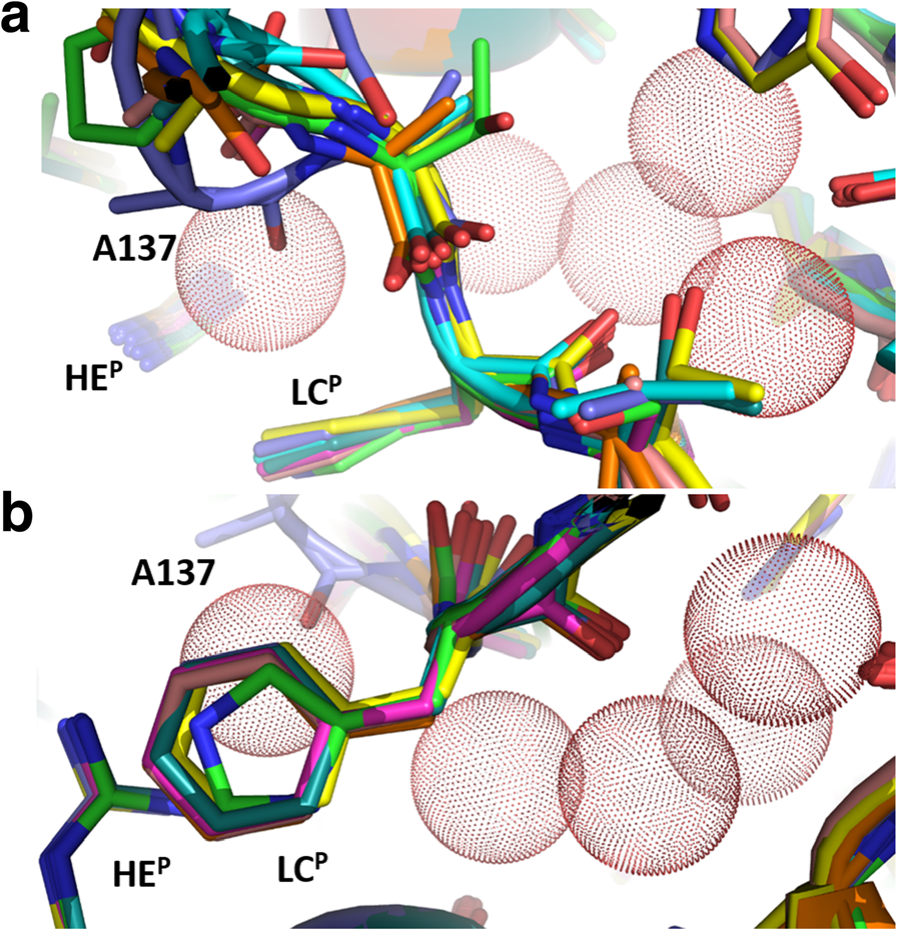
AQP8 models are compatible with existence of a water filled side pore beneath loop C. **a** Top view of loop C in aligned AQP8 models and *At*TIP2;1 with amino acid residues shown as sticks and oxygens of waters in the side pore of *At*TIP2;1 represented by dotted spheres. Although the monomeric models were generated without waters, there is no obvious clash with the four water molecules in the side pore. **b** Side view of the same region. The water molecule connecting the arginine at HE^P^ to a carbonyl oxygen in loop C can be accommodated in all models except *Hs*AQP8. In human AQP8 this residue (A137) sits deeper and overlaps with the oxygen of the water from *At*TIP2;1. Thereby it blocks a potential connection of the side pore to the main pore in this model. *At*TIP2;1 – green, *Hs*AQP8 – slate blue, *Bt*AQP8 – cyan, *Rn*AQP8 – orange, *Fp*AQP8 – magenta, *Cp*AQP8 – greenblue*, Xt*AQP8 – yellow, *Ss*AQP8b – salmon

In all solved AQP structures except *At*TIP2;1 the arginine at HE^P^ is stabilized by hydrogen bonding to a backbone carbonyl of the C loop. In *At*TIP2;1 the arginine is also connected to the C loop but indirectly via hydrogen bonding a water molecule [22]. This water molecule is located at the possible entrance of a water filled side pore going under loop C and up to the surface of the protein. The waters in the side pore have been speculated to form a proton wire that may assist in deprotonation of ammonium ions leading to an increased local concentration of ammonia and thereby elevating the net permeation of ammonia. So far there is no experimental evidence for such a mechanism, and it is conceivable that these water molecules instead have a strictly structural role in the protein. Interestingly, there is no clash between the four water molecules in the side pore of *At*TIP2;1 and the residues of the modelled AQP8s (Fig. 12). Thus the models are compatible with a water filled side pore in this region and all static models, except *Hs*AQP8, are compatible with a water molecule connecting the arginine at HE^P^ with loop C in a *At*TIP2;1-like fashion. In *Hs*AQP8 the afore mentioned carbonyl of the C loop adopts a position found in e.g. water specific AQPs, and the carbonyl clashes with the water at this location in *At*TIP2;1 (Fig. 12). A possible explanation of this difference is that the *Hs*AQP8 model deviates from the other models at this site because a larger part of its C loop was initially modelled based on the water specific *Ec*AqpZ. Nonetheless, the MD simulation with *Hs*AQP8 demonstrates formation of a *At*TIP2;1-like hydrogen bond network that connects the C loop via an exchangeable water molecule to the arginine at HE^P^. Furthermore, the MD simulation shows that the water filled side pore persists over time even though the positions of water molecules are not fixed. Thus a functional role of a side pore in AQP8, possibly in deprotonation, cannot be excluded.

The surface electrostatics of AQP8 may hold clues to its orientation in membranes. The surface close to the selectivity filter is more negative than the opposite side where N and C termini are protruding. This may indicate an evolutionary selection based on stability at a lower pH. Alternatively, the distribution of charges can be a result of the requirements for membrane insertion, as suggested by the positive-inside rule [38]. This rule might apply when AQP8 is localized to the plasma membrane, however, when nuclear encoded proteins, like AQP8, are localized to the mitochondria they may not always follow this rule. Nevertheless, depending on the insertion pathway, negatively charged residues have been reported to be under-represented on the matrix side of the membrane [39]. This would suggest that the N and C termini of mitochondrial AQP8s are positioned on the matrix side. In both cases, the more acidic side of AQP8s and the putative side pore is facing the more acidic intermembrane space.

## Conclusions

Overall the models of the selected AQP8s are compatible both with an *At*TIP2;1-like selectivity filter and the existence of a side pore. Using the published *At*TIP2;1 X-ray structure as a template, the presented AQP8 models are likely to be closer to the real structures as compared with currently available AQP8 models in the MIPmodDB [33]. The models generated in this study provide a base for further detailed structural analysis. These may include cross-linking experiments, which can be analyzed by mass spectrometry. Alternatively, FRET spectrometry is a known tool to derive distances between neighboring residues, which are accordingly labeled. Comparison of functional data from mutational studies or between different isoforms may lead to insights in functional mechanisms. As previously shown for other AQPs, the presented tetrameric model of human AQP8 may be used to find drug binding sites or improve potential binders by interaction mapping.

## Methods

### Mapping structural conservation

To map the structural conservation of AQPs, 49 structures identified by a BLAST search with the sequence of *Hs*AQP8 as query were retrieved from PDB, leaving out the low resolution structures 3IYZ and 3J41. PDB IDs are given in Additional file 6.

The structural alignment was calculated in PyMOL [40] with the Python script color_by_conservation written by Jason Vertrees. The scores were recalculated as 1 minus original score.

All structural images in this article were made with PyMOL [40].

### Homology modeling

A flowchart is summarizing the main steps of the process is presented in Additional file 7: Figure S5. Homology modeling was performed using SWISS-MODEL workspace [41] running in project mode. Accession numbers for the modelled proteins are given in Table 1. An initial multiple sequence alignment was created for all models together with *At*TIP2;1 by Clustal Omega (v1.2.0) [42] and modified in loop C to match LC^P^ phenylalanines to histidine 131 from *At*TIP2;1 using Discovery Studio Visualizer (Accelrys Software Inc). A phylogenetic tree was calculated in MEGA [43] using Neighbor-Joining algorithm [44]. The distances are computed by the JTT matrix-based method [45]. Final pair-wise alignments of template and model sequences were saved as projects in Deep-View (v4.1.0) [46] available under http://www.expasy.org/spdbv/. In comparison, an automatic sequence alignment with *Ec*AqpZ placed S118 instead of N119 at LC^P^ (Fig. 2). As the HE^P^ arginine in *Ec*AqpZ is oriented towards the pore, aligning phenylalanine of AQP8s in LC^P^ would cause a steric clash. This part of the structures is later taken from the *At*TIP2;1-based models (PDB ID 5I32). To increase the chance of achieving models based on *Ec*AqpZ (PDB ID 1RC2), a smaller residue of AQP8s was allowed to be aligned with N119 of *Ec*AqpZ. The alternative models were used to create chimeras mainly consisting of structural parts based on the *At*TIP2;1 template supplemented with parts modelled on *Ec*AqpZ (Table 1). Notably, the position of the residue at LC^P^ in the final hybrid models comes from models originating from *At*TIP2;1. The chains of the hybrid models were connected in Coot [47]. All resulting models were energy minimized based on GROMOS96 [48] force field embedded in Deep-View (> 400 rounds). Additionally, the backbone at LE^P^ carbonyls were energy minimized in NAMD (> 5000 rounds) [49].

### Validation and analysis of monomeric models

Energy minimized models were stereochemically improved utilizing Coot [47] and validated by MolProbity [50], both included in Phenix package [51]. This cycle was repeated until energy, stereochemistry and structural features were acceptable and did not appear to improve further.

The diameter of the pore was estimated using the program HOLE [52].

### MD simulation

A tetramer of *Hs*AQP8 was constructed in PyMOL [40] based on the 4-fold symmetry axis of *At*TIP2;1 coinciding with the central pore. Protonation states of amino acid side chains were assigned as predicted by PROPKA3 [32]. Crystallographically resolved waters derived from the *At*TIP2;1 structure [22] were added, including water molecules positioned in the membrane spanning monomeric pore, and the positions were energy minimized with > 2000 steps using the steepest decent algorithm in NAMD [49], with everything fixed except the added water molecules. The protein, including the water molecules from the previous step, were positioned in a 115 Å × 115 Å 1-palmitoyl-2-oleoyl-*sn*-glycero-3-phosphocholine (POPC) membrane made with the membrane builder function in VMD [53], and then solvated with the TIP3P water model [54] to a resulting lipid/water/protein box of 115 × 115 × 85 Å. The charges in the system were neutralized by addition of sodium and chloride ions. Lipids and waters from the membrane patch were removed, if overlapping or being closer to the protein than 0.8 Å for lipids and 1.2 Å for waters. The resulting system consisted of 94,254 atoms and was used in all subsequent equilibrations and MD runs, applying the CMAP corrected CHARMM22 force field [55].

To equilibrate the membrane to the *Hs*AQP8 and vice versa before MD runs, an initial 10,000 steps of conjugated gradient minimization was performed on the system with the protein, ions and crystallographically resolved waters from the *At*TIP2;1 structure fixed, the remainder of waters restrained, and with the phosphate of the POPC head groups restrained in the z-axis direction.

This was followed by > 250 ps of MD equilibration of the system with the protein and crystallographically resolved waters (derived from the *At*TIP2;1 structure) fixed and the phosphate of the POPC molecules restrained in the z-axis direction (normal to the lipid bilayer plane) in the NPT ensemble with periodic boundary conditions (PBC) and long-range electrostatics included with the Particle mesh Ewald method (PME) [56], with grid spacing set to 1. Langevin dynamics was applied with Langevin damping coefficient set to 0.5 per ps, which was gradually increased to 1 during the run. The temperature was set to 310 K.

This equilibration run allowed the lipid “tails” to adapt fully to the protein surface and to adapt randomized positions in relation to each other. The above melting of the lipid “tails” was followed by 250 ps equilibration in the NVT ensemble with PBC and PME, at 310 K. Water molecules in the pore were subjected to harmonic restraints in the x,y,and z direction. Furthermore, the phosphorous atom in the lipid head groups where restrained in the z-axis direction. During the time frame of the equilibration, harmonic restraints were released gradually. The protein was initially fixed, followed by harmonic restraints of the whole protein, and then slow release of first the sidechain and later the backbone from the restraints.

The equilibrated systems were then simulated for 3 ns in a NPzAT ensemble at 310 K and 1 atm of pressure. All simulations were done with the timestep set to 1 per fs, PME, PBC and Langevin damping coefficient set to 1 per ps.

## Additional files

Additional file 1: **Figure S1.** Phylogenetic tree of protein sequences of AQP8 models and the used templates. The scheme is congruent with the species tree with two exceptions. First, AQP8 from frog (*Xt*AQP8) groups with mammalian AQP8s, instead of being basal to amniota and second, the distance of human AQP8 (*Hs*AQP8) to rat AQP8 (*Rn*AQP8) is longer than to bovine AQP8 (*Bt*AQP8). Scale is shown in amino acid substitutions per position leading to a branch length sum of 3.5. (TIFF 1987 kb)

Additional file 2: **Figure S2.** Cartoon representation highlighting parts of hybrid models that are based on the structure of *Ec*AqpZ. All seven final monomeric composite models are aligned, and regions that are initially modelled on *Ec*AqpZ are marked in magenta. Residues at the five positions of the selectivity filter of *Hs*AQP8, as well as the phenylalanine (F85 in *Hs*AQP8) that occludes the pore in models solely relying on *At*TIP2;1, are depicted as sticks. The extent of the *Ec*AqpZ based structure in the chimeric models varies but as a minimum consist of the complete helix 2 (H2) and the consecutive residues up to and including the phenylalanine corresponding to F85 in *Hs*AQP8. (TIFF 703 kb)

Additional file 3: **Figure S3.** Structural alignment of AQP8 models. The seven AQP8 models and the main template *At*TIP2;1 are shown in ribbon representation, and the five residues of the selectivity filters are depicted as sticks. The highest variability is found in loop regions, especially in loop C. Still the part of the loop contributing to the selectivity filter is consistently modelled in to a *At*TIP2;1-like structure. a Side view of models and the template. b View of opposite side relative (a). c Top view of the selectivity filter. *At*TIP2;1 – green, *Hs*AQP8 – slate blue, *Bt*AQP8 – cyan, *Rn*AQP8 – orange, *Fp*AQP8 – magenta, *Cp*AQP8 – greenblue*, Xt*AQP8 – yellow, *Ss*AQP8b – salmon. (TIFF 883 kb)

Additional file 4: **Figure S4.** RMSD of C_α_ *Hs*AQP8 in MD simulation support a conformational equilibrium at the end of the simulation. The tetrameric model of *Hs*AQP8 was gradually equilibrated and released during the first ns, after which the MD simulation was run for 3 ns. The RMSD levels out around 2.3 Å relative to the starting position, indicating that a stable conformation was reached. (PNG 99 kb)

Additional file 6: Text. The PDB IDs of the structures used for the alignment. 1FQY, 1FX8, 1H6I, 1IH5, 1J4N, 1LDA, 1LDF, 1LDI, 1RC2, 1SOR, 1YMG, 1Z98, 2ABM, 2B5F, 2B6O, 2B6P, 2C32, 2D57, 2EVU, 2F2B, 2O9D, 2O9E, 2O9F, 2O9G, 2W1P, 2W2E, 2ZZ9, 3C02, 3CLL, 3CN5, 3CN6, 3D9S, 3GD8, 3LLQ, 3M9I, 3NE2, 3NK5, 3NKA, 3NKC, 3ZOJ, 4CSK, 4IA4, 4JC6, 4NEF, 4OJ2, 5BN2, 5C5X, 5DYE, 5I32. (DOCX 12 kb)

Additional file 7: **Figure S5.** Modeling process. Data presented in sharp edged parallelograms and processes with round edged shapes. Each of seven vertebrate AQP8s was modelled twice, using *At*TIP2;1 and *Ec*AqpZ as template. Resulting model pairs were hybridized and further refined. Based on the final monomeric model a tetrameric model was formed for *Hs*AQP8, and its stability was studied in MD simulations. (TIFF 118 kb)

## Abbreviations

AQP: Aquaporin
*At*: *Arabidopsis thaliana*
*Bt*: *Bos taurus*
*Cp*: *Chrysemys picta bellii*
*Ec*: *Escherichia coli*
*Fp*: *Falco peregrinus*
H2^P^: Helix 2 position
H5^P^: Helix 5 position
HE^P^: Helix E position
*Hs*: *Homo sapiens*
LC^P^: Loop C position
LE^P^: Loop E position
MD: Molecular dynamics
MIP: Major intrinsic protein
PBC: Periodic boundary conditions
PME: Particle mesh Ewald method
POPC: 1-palmitoyl-2-oleoyl-*sn*-glycero-3-phosphocholine
*Rn*: *Rattus norvegicus*
*Ss*: *Salmo salar*
*Ta*: *Triticum aestivum*
TIP: Tonoplast intrinsic protein
*Xt*: *Xenopus tropicalis*
