# Supplementary figures and images for: A structural preview of aquaporin 8 via homology modeling of seven vertebrate isoforms
Source: BMC Struct Biol. 2018 Feb 17;18:2. doi: 10.1186/s12900-018-0081-8 (PMC5816522; doi:10.1186/s12900-018-0081-8)

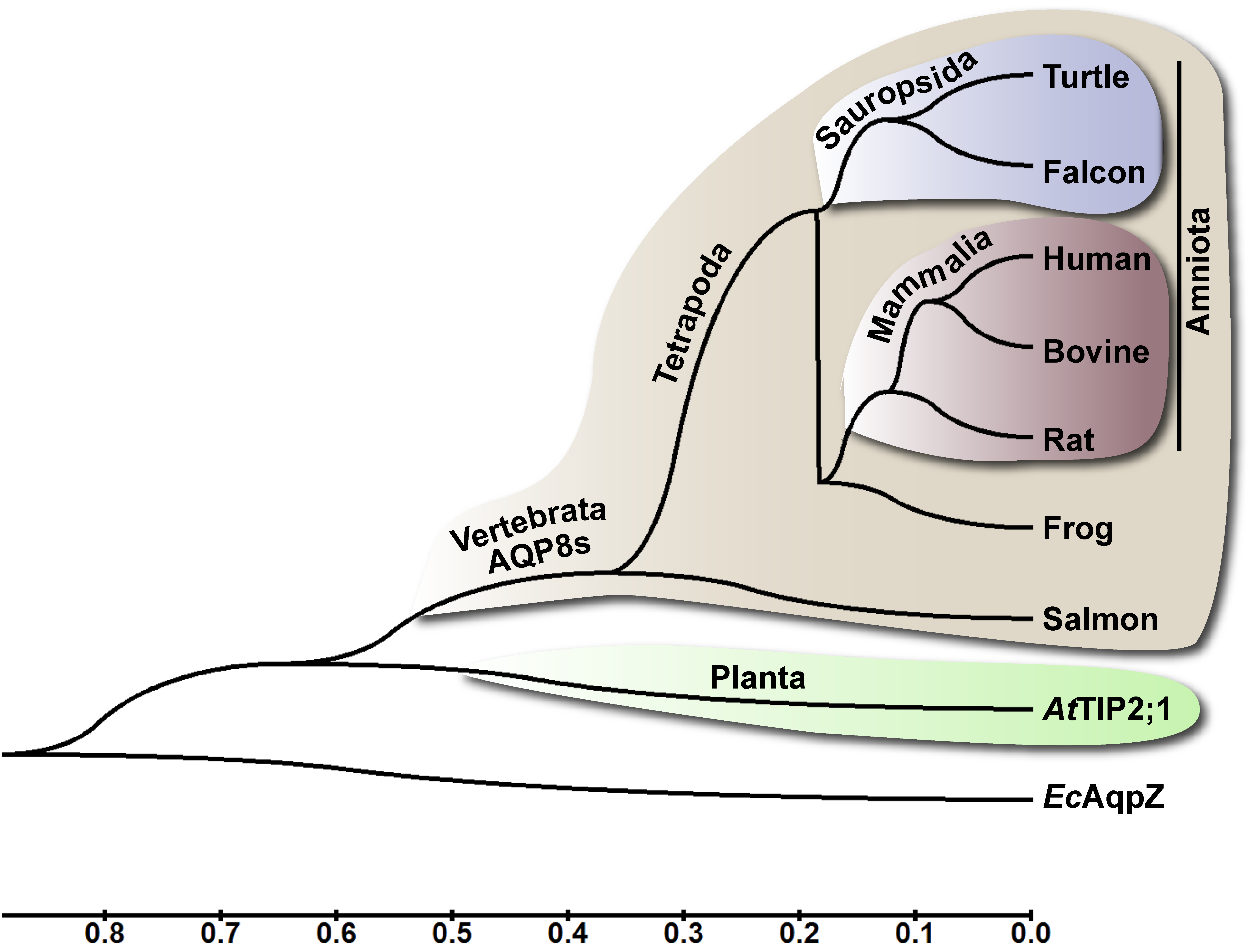

Supplement: Supplementary file 2 — Figure S1. Phylogenetic tree of protein sequences of AQP8 models and the used templates. The scheme is congruent with the species tree with two exceptions. First, AQP8 from frog (XtAQP8) groups with mammalian AQP8s, instead of being basal to amniota and second, the distance of human AQP8 (HsAQP8) to rat AQP8 (RnAQP8) is longer than to bovine AQP8 (BtAQP8). Scale is shown in amino acid substitutions per position leading to a branch length sum of 3.5. (TIFF 1987 kb) [file 12900_2018_81_MOESM1_ESM.tif]

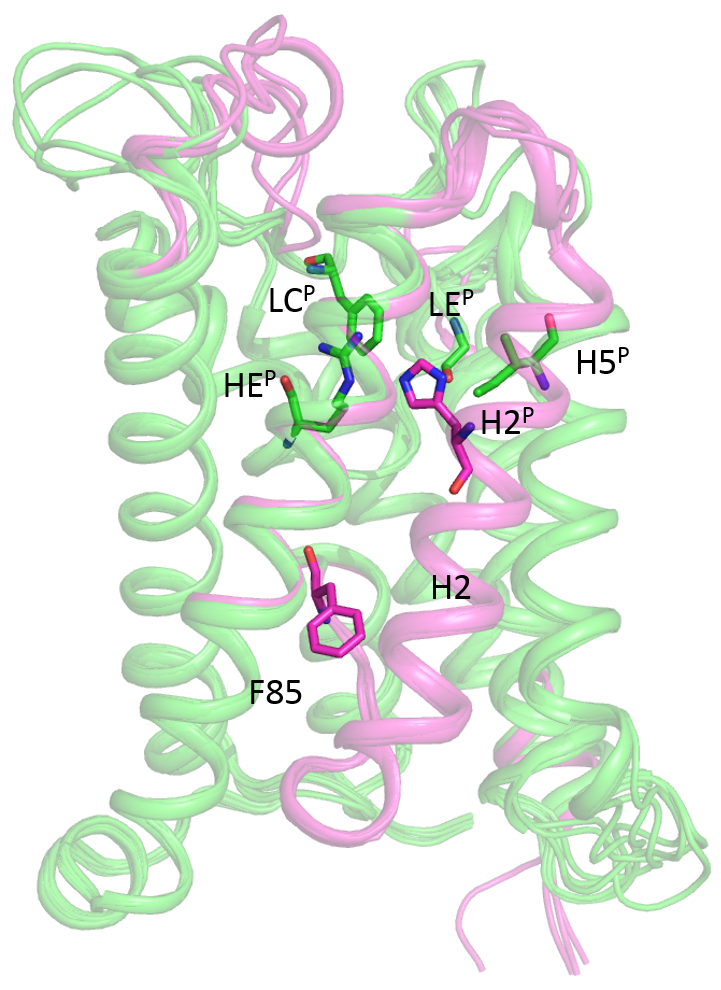

Supplement: Supplementary file 3 — Figure S2. Cartoon representation highlighting parts of hybrid models that are based on the structure of EcAqpZ. All seven final monomeric composite models are aligned, and regions that are initially modelled on EcAqpZ are marked in magenta. Residues at the five positions of the selectivity filter of HsAQP8, as well as the phenylalanine (F85 in HsAQP8) that occludes the pore in models solely relying on AtTIP2;1, are depicted as sticks. The extent of the EcAqpZ based structure in the chimeric models varies but as a minimum consist of the complete helix 2 (H2) and the consecutive residues up to and including the phenylalanine corresponding to F85 in HsAQP8. (TIFF 703 kb) [file 12900_2018_81_MOESM2_ESM.tif]

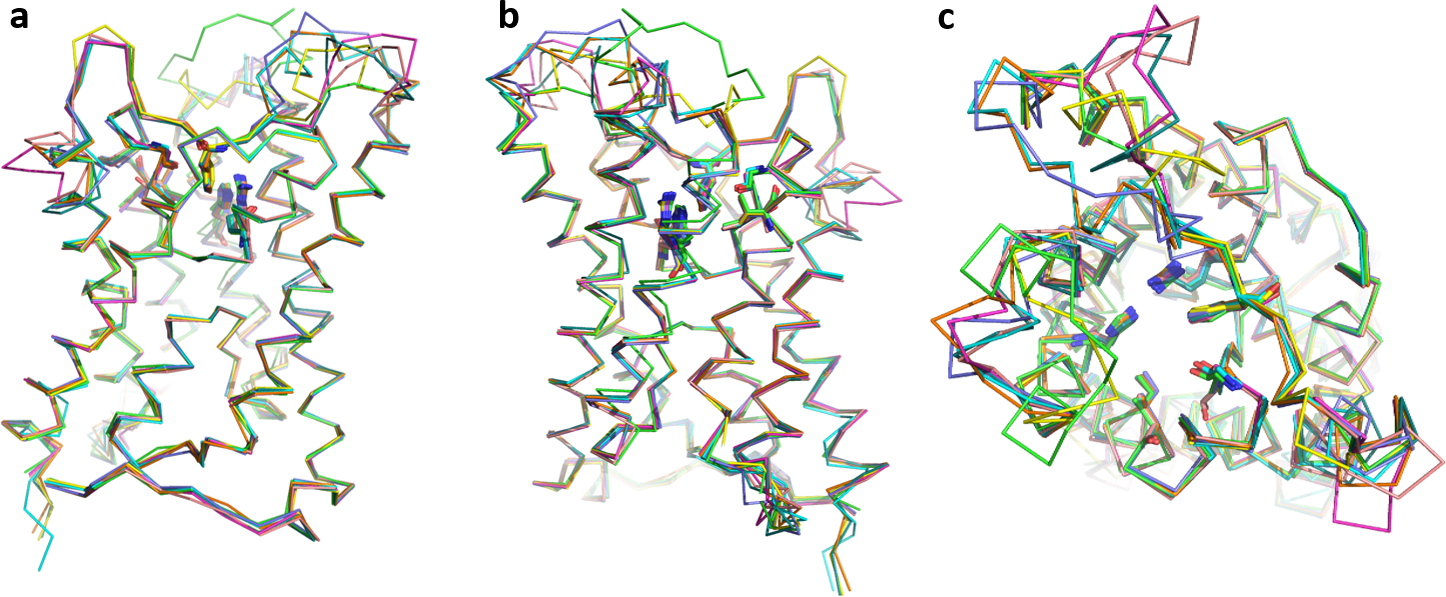

Supplement: Supplementary file 4 — Figure S3. Structural alignment of AQP8 models. The seven AQP8 models and the main template AtTIP2;1 are shown in ribbon representation, and the five residues of the selectivity filters are depicted as sticks. The highest variability is found in loop regions, especially in loop C. Still the part of the loop contributing to the selectivity filter is consistently modelled in to a AtTIP2;1-like structure. a Side view of models and the template. b View of opposite side relative (a). c Top view of the selectivity filter. AtTIP2;1 – green, HsAQP8 – slate blue, BtAQP8 – cyan, RnAQP8 – orange, FpAQP8 – magenta, CpAQP8 – greenblue, XtAQP8 – yellow, SsAQP8b – salmon. (TIFF 883 kb) [file 12900_2018_81_MOESM3_ESM.tif]

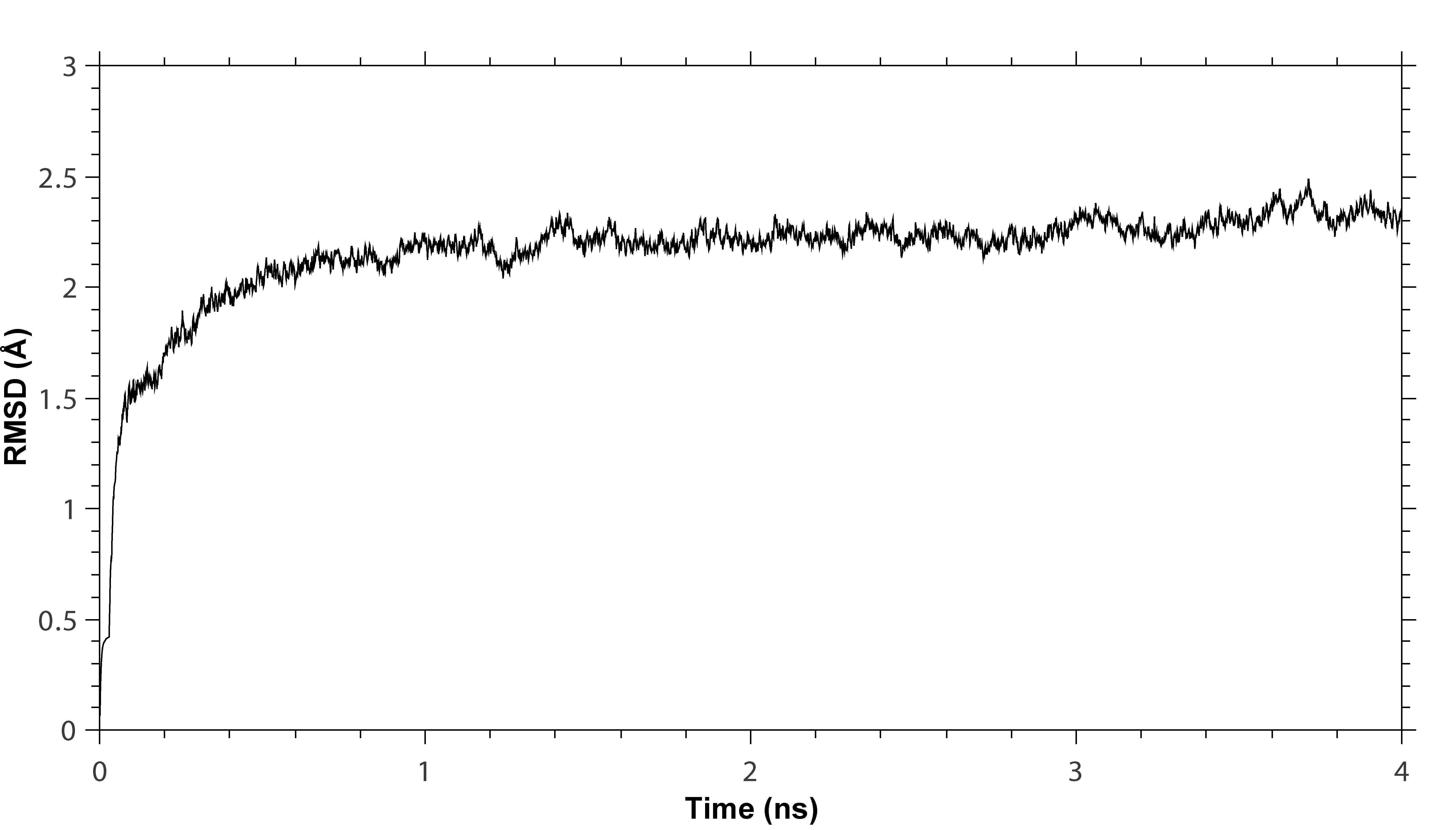

Supplement: Supplementary file 5 — Figure S4. RMSD of Cα HsAQP8 in MD simulation support a conformational equilibrium at the end of the simulation. The tetrameric model of HsAQP8 was gradually equilibrated and released during the first ns, after which the MD simulation was run for 3 ns. The RMSD levels out around 2.3 Å relative to the starting position, indicating that a stable conformation was reached. (PNG 99 kb) [file 12900_2018_81_MOESM4_ESM.png]

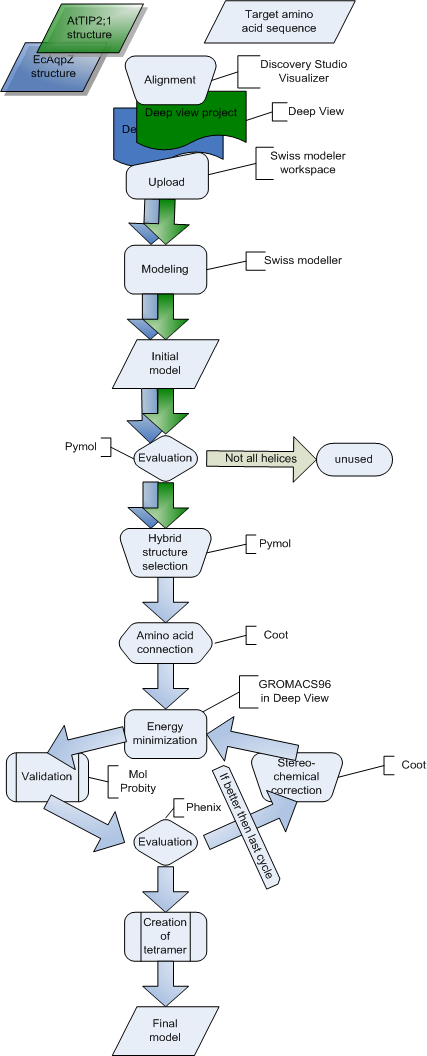

Supplement: Supplementary file 7 — Figure S5. Modeling process. Data presented in sharp edged parallelograms and processes with round edged shapes. Each of seven vertebrate AQP8s was modelled twice, using AtTIP2;1 and EcAqpZ as template. Resulting model pairs were hybridized and further refined. Based on the final monomeric model a tetrameric model was formed for HsAQP8, and its stability was studied in MD simulations. (TIFF 118 kb) [file 12900_2018_81_MOESM7_ESM.tif]
