# Supplementary material for: A structural preview of aquaporin 8 via homology modeling of seven vertebrate isoforms
Source: BMC Struct Biol. 2018 Feb 17;18:2. doi: 10.1186/s12900-018-0081-8 (PMC5816522; doi:10.1186/s12900-018-0081-8)
Supplement: Supplementary file 6 — Text. The PDB IDs of the structures used for the alignment. 1FQY, 1FX8, 1H6I, 1IH5, 1J4N, 1LDA, 1LDF, 1LDI, 1RC2, 1SOR, 1YMG, 1Z98, 2ABM, 2B5F, 2B6O, 2B6P, 2C32, 2D57, 2EVU, 2F2B, 2O9D, 2O9E, 2O9F, 2O9G, 2W1P, 2W2E, 2ZZ9, 3C02, 3CLL, 3CN5, 3CN6, 3D9S, 3GD8, 3LLQ, 3M9I, 3NE2, 3NK5, 3NKA, 3NKC, 3ZOJ, 4CSK, 4IA4, 4JC6, 4NEF, 4OJ2, 5BN2, 5C5X, 5DYE, 5I32. (DOCX 12 kb) [file 12900_2018_81_MOESM6_ESM.docx]

### Additional file 2: Text. The PDB IDs of the structures used for the alignment.

1FQY, 1FX8, 1H6I, 1IH5, 1J4N, 1LDA, 1LDF, 1LDI, 1RC2, 1SOR, 1YMG, 1Z98, 2ABM, 2B5F, 2B6O, 2B6P, 2C32, 2D57, 2EVU, 2F2B, 2O9D, 2O9E, 2O9F, 2O9G, 2W1P, 2W2E, 2ZZ9, 3C02, 3CLL, 3CN5, 3CN6, 3D9S, 3GD8, 3LLQ, 3M9I, 3NE2, 3NK5, 3NKA, 3NKC, 3ZOJ, 4CSK, 4IA4, 4JC6, 4NEF, 4OJ2, 5BN2, 5C5X, 5DYE, 5I32
